# Characterization of the complete chloroplast genome of traditional Chinese herb, *Solanum japonense* Nakai. (Solanaceae)

**DOI:** 10.1080/23802359.2020.1860713

**Published:** 2021-01-19

**Authors:** Xiaoxia Zhang, Xiaofeng Zhang, Huan Wang, Shilong Chen, Tingfeng Cheng, Shengbo Shi, Dangwei Zhou

**Affiliations:** a College of Medicine, Xi'an International University, Xi’an, P. R. China; b Key Laboratory of Adaptation and Evolution of Plateau Biota (AEPB), NorthwestInstitute of Plateau Biology, Chinese Academy of Sciences, Xining, P. R. China

**Keywords:** *Solanum japonense* Nakai., chloroplast genome, phylogenetic analysis

## Abstract

*Solanum japonense* Nakai. (Solanaceae) is a traditional Chinese medicine and animal fodder in Asian continent. Here, the complete chloroplast genome sequence of *S. japonense* was determined by the Illumina Hiseq technology. The complete chloroplast genome of *S. japonense* was 155,415 bp and the GC content was 37.81%. The typical circular quadripartite structure was composed with two inverted repeat (IR) regions with 25,588 bp, a large single-copy (LSC) region (85,931 bp) and a small single-copy (SSC) region (18,344 bp). The chloroplast genome of *S. japonense* contained 132 unigenes, which contained 86 protein-coding genes, 37 tRNA and 8 rRNA genes and one pseudo-gene. Moreover, 183 SSRs were identified and 65% (119)of them located at LSC region. A Maximum-Likelihood (ML) phylogenetic analysis based on chloroplast genomes indicated that *S. japonense* was closely related to *S.dulcamara*, *S.nigrum*. Our results would provide a valuable resource for resource utilization and the phylogenetic studies of species of Dulcamara sect. in Solanaceae.

*Solanum japonense* Nakai. (Yehaiqie in Chinese) is a widely perennial herb, belongs to Solanaceae family, which distributes in wastelands, slopes or forests ranged 300 m to 2900 m in China (Flora in China 1979). This species and *Solanum dulcamara* both belongs to Sect. Dulcamara (Moench), while the former has deltate, broadly lanceolate or ovate-lanceolate leaves, often with 2 basal lobes (Flora in China 1979). As its vine, flower and fruits were similar to *S. dulcamara*, it is often confused with *S. dulcamara* and to treat rheumatism and skin diseases in Asia and India (Li 2004; Kumar et al. 2009; Yuan et al. 2020). Solanaceae has various alkaloids and is an important medicine resource. Recently, the study also showed *S. dulcamara* and other related species were abundant in terpenoids and has various pharmacological activities (Yuan et al. 2020). As the important wild plant resource, *S.japonense* taxon and pharmacological traits have been paid attention (Flora in China 1979; Li 2004), however there still known limit on chloroplast genome of this species till now. In this paper, we sequenced and assembled the complete chloroplast of *S. japonense* using Illumina Hiseq platform. The cp genome was annotated and submitted to the Genbank (Accession number: MW077727).

We collected fresh leaves from a single individual of this species from Minhe County (102.733°, 36.143°, 2809 m), Qinghai, China, and dried leaves with silica gel. Voucher speciements were deposited in the herbarium of Northwest Institute of Plateau Biology, CAS (HNWP, Zhou2020024). Total DNA was extracted from the fresh leaves with the DNeasy Plant MiniKit (QIAGEN, CA, USA) according to the manufacturer’s instructions. DNA quality was assessed based on spectrophotometry and electrophoresis in 1% (w/v) agarose gel, and then the good integrity and purity DNA was used for library construction and sequencing. The average insertion of the library was 320 bp and whole genome sequencing with 150 bp pair-end reads using the Illumina Hiseq platform (San Diego CA, USA) at Genepioneer Biotechnologies Inc., Nanjing, China.

In total, we obtained about 17, 793,213 high quality clean reads and QC30 value was 93.96%. The cp genome was assembly using NovoPlasty software (Dierckxsens et al. 2016) and the previously published cp genome of *S. dulcamara* (Amiryousefi et al. 2018) was used as seed reference. We visualized the genome by Geneious version8.05 (Kearse et al. 2012). Gene annotation firstly perform with DOGMA (Wyman et al. 2004) and CpGAVAS (Liu et al. 2012), then corrected manually with the Geneious (Kearse et al. 2012). Finally, the physical map of cp genome of *S. japonense* was done with CpGAVAS (Liu et al. 2012).The complete cp genome sequence and its annotations were submitted to Genbank (MW077727).

The complete chloroplast genome of *S. japonense* is 155,415 bp in size and the average GC content is 37.81%. In the typical circular quadripartite structure, there was a pair of inverted repeat (IR) regions with 25,588 bp in length, which separated by a large single-copy (LSC) region (85,931 bp) and a small single-copy (SSC) region (18,344 bp).The chloroplast genome of *S. japonense* contained 132 unigenes, which was composed of 86 protein-coding genes, 37 tRNA and 8 rRNA genes and one psedo-gene. Moreover, 183 SSRs were identified and 65% (119) of them existed in LSC region. Compared the four cp gnome of Solanaceae, most genes were conserved and the variation displayed in the intergeneric regions.

To identified the phylogenetic relationship of *S. japonense* and other 39 species of Solanaceae, the whole chloroplast genome of them were aligned with MAFFT software (Katoh and Standley 2013), and species of *Scrophularia dentata* was set as the outgroup species. Maximum -Likelihood (ML) phylogenetic analysis was conducted adopting HKY85 model by PhyML3.0 (Guindon et al. 2010) with 100 bootstrap replicates. The phylogenetic tree displayed that *S. japonense* was closer clustered with *S. dulcamara* while *S.nigrum* was out of them clearly (Figure 1). Our cp genome data of *S. japonense* would facilitate population, genetic identification and cp genetic engineering research of this traditional Chinese herb in the future.

**Figure 1.**
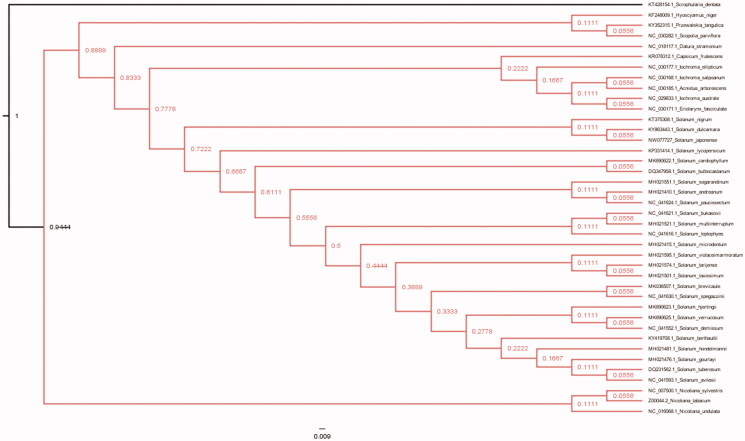
Maximum-Likelihood (bootstrap repeat is 100) phylogenetic trees of *S. japonense* and related species of Solanaceae based on the whole cp genomes. Chloroplast genomes: GenBank accession numbers: *Solanum japonense* **(MW077727; this study)**, *Solanum gourlayi* (MH021476.1), *Solanum avilesii* (NC_041593.1), *Solanum hondelmamii* (MH021481.1), *Solanum hondelmamii* (NC_041608.1), *Solanum berthaultii* (KY419708.1), *Solanum tuberosum* (DQ231562.1), *Solanum violaceimarmoratum* (MH021595.1), *Solanum laxissimum* (MH021501.1), *Solanum tarijense* (MH021574.1), *Solanum demissum* (NC_041552.1), *Solanum hjertingii* (MK690623.1), *Solanum verrucosum* (MK690625.1), *Solamum microdontum* (MH021415.1), *Solanum spegazzinii* (NC_041630.1), *Solanum brevicaule* (MK036507.1), *Solanum leptophyes* (NC_041616.1), *Solanum bukasovii* (NC_041621.1), *Solanum multiinterruptum* (MH021521.1), *Solanum sogarandinum* (MH021551.1), *Solanum paucissectum* (NC_041624.1), *Solanum andreanum* (MH021410.1), *Solanum cardiophyllum*(MK690622.1), *Solanum bulbocastanum*(DQ347958.1), *Solanum lycopersicum* (KP331414.1), *Solanum dulcamara*(KY863443.1), *Solanum nigrum* (KT375308.1), *Scrophularia dentate* (KT428154), *Acnistus arborescens* (NC_030185.1), *Capsicum frutescens* (KR078312.1), *Datura stramonium* (NC_018117.1), *Eriolarynx fasciculate* (NC_030171.1), *Hyoscyamus niger* (KF248009.1), *Iochroma australe* (NC_029833.1), *Iochroma ellipticum* (NC_030177.1), *Iochroma salpoanum* (NC_030168.1), *Nicotiana sylvestris* (NC_007500.1), *Nicotiana tabacum* (Z00044.2), *Nicotiana undulata* (NC_016068.1), *Solanum bulbocastanum cultivar* PT29 (DQ347958.Q), *Scopolia parviflora* (NC_030282.1).

## Data Availability

*Solanum_japonense_*N cp genome data was submitted to Genbank ( accession number: MW077727).
